# Cloning and expression characterization of elongation of very long-chain fatty acids protein 6 (*elovl6*) with dietary fatty acids, ambient salinity and starvation stress in *Scylla paramamosain*

**DOI:** 10.3389/fphys.2023.1221205

**Published:** 2023-07-12

**Authors:** Zhideng Lin, Zhouyu Wu, Chaoyang Huang, Huangbin Lin, Mingyao Zhang, Mingfeng Chen, Kunhuang Han, Weiqing Huang, Shaojiang Ruan

**Affiliations:** ^1^ College of Life Science, Ningde Normal University, Ningde, China; ^2^ Engineering Research Center of Mindong Aquatic Product Deep-Processing, Ningde Normal University, Ningde *,* China

**Keywords:** ELOVL6, fatty acids, salinity stress, starvation stress, *Scylla paramamosain*

## Abstract

**Introduction:** Elongation of very long-chain fatty acids protein 6 (ELOVL6) played crucial roles in regulating energy expenditure and fatty acid metabolism. Many studies have performed to investigate the physiological roles and regulatory mechanisms of *elovl6* in fish and animals, while few studies were reported in crustaceans.

**Methods:** Here we reported on the molecular cloning, tissue distribution and expression profiles in response to dietary fatty acids, ambient salinity and starvation stress in *Scylla paramamosain* by using rapid amplification of cDNA ends (RACE) and quantitative real-time PCR.

**Results:** Three *elovl6* isoforms (named *elovl6a, elovl6b* and *elovl6c*) were isolated from *S. paramamosain* in the present study. The complete sequence of *elovl6a* was 1345 bp, the full-length sequence of *elovl6b* was 1419 bp, and the obtained *elovl6c* sequence was 1375 bp in full length. The *elovl6a, elovl6b* and *elovl6c* encoded 287, 329 and 301 amino acids respectively, and exhibited the typical structural features of ELOVL protein family members. Phylogenetic analysis showed that the ELOVL6a from *S. paramamosain* clustered most closely to ELOVL6 from *Portunus trituberculatus* and *Eriocheir sinensis*, while the ELOVL6b and ELOVL6c from *S. paramamosain* gathered alone into a single branch. Quantitative real-time PCR exhibited that the relatively abundant expression of *elovl6b* was observed in intestine and stomach, and the *elovl6a* and *elovl6c* were highly expressed in hepatopancreas. In addition, studies found that replacing fish oil with soybean oil could significantly increase the transcriptional levels of three *elovl6* in hepatopancreas of *S. paramamosain*, and the expression of *elovl6a* and *elovl6c* in hepatopancreas were more sensitive to dietary fatty acids than the *elovl6b*. Compared with the normal sea water group (27‰), the expression of sterol-regulatory element binding protein1c *(srebp-1), elovl6a, elovl6b* and *elovl6c* were upregulated in the low salinity groups, particularly in 7‰. On the contrary, the starvation stress suppressed the expression of *srebp-1, elovl6a, elovl6b* and *elovl6c*.

**Discussion:** These results may contribute to understand the functions of *elovl6* in fatty acid synthesis and regulatory mechanisms in crustaceans.

## 1 Introduction

The synthesis of long chain fatty acids (LCFAs) *de novo* was accomplished by elongation and desaturation steps (Green et al., 2010; Xie et al., 2021). As rate-limiting enzymes, elongation of very long-chain fatty acids proteins (ELOVL) were responsible for catalyzing the elongation step, which can elongate two carbons to pre-existing fatty acyl chains (Green et al., 2010; Guillou et al., 2010). The ELOVL family was divided into seven members in mammals based on different catalytic substrates and sequence characterization (Jakobsson et al., 2006). Generally, ELOVL5, ELOVL4 and ELOVL2 were inclined to elongate polyunsaturated fatty acids (PUFA), while ELOVL7, ELOVL6, ELOVL3 and ELOVL1 preferred to catalyze monounsaturated fatty acids (MUFA) and saturated fatty acids (SFA) (Castro et al., 2016). As a final elongase participated in LCFAs *de novo*, the ELOVL6 was first reported in mice, which showed the functions of elongating palmitoleic acid (C16:1n-7) and palmitate (C16:0) to vaccenci acid (C18:1n-7) and stearate (C18:0) respectively (Moon et al., 2001; Matsuzaka et al., 2002; Shi et al., 2017). Recently, numerous studies have been investigated to determine the physiological roles and regulatory mechanisms of *elovl6* in mammals (Matsuzaka et al., 2007; Saito et al., 2011; Tan et al., 2015; Bae et al., 2016; Su et al., 2018). By contrast, the roles of *elovl6* in aquatic animals was still unclear, which only reported in *Larimichthys crocea* (Li et al., 2019), *Misgurnus anguillicaudatus* (Chen et al., 2018), *Oncorhynchus mykiss* (Li et al., 2020) and *Eriocheir sinensis* (Shi et al., 2016).

The *elovl6* is mainly expressed in lipogenic tissues and is closely associated with metabolic diseases (like atherogenesis, insulin resistance and hepatic inflammation) and energy balance (Matsuzaka et al., 2007; Matsuzaka and Shimano, 2009; Takashi et al., 2012; Motoko et al., 2015; Tan et al., 2015; Zhao et al., 2017; Nakamura et al., 2018; Su et al., 2018). Previous studies have shown that the expression of *elovl6* was sensitive to nutrients (Matsuzaka et al., 2002; Leroux et al., 2016; Shi et al., 2016; Li et al., 2019), environmental factors (Tan et al., 2015; Chen et al., 2018) and hormonal (Matsuzaka et al., 2007; Matsuzaka and Shimno, 2009; Sun et al., 2013; Li et al., 2020). In mammals, transcription of *elovl6* was regulated by the transcription factors such as carbohydrate response element binding protein (CHREB), sterol-regulatory element binding protein-1c (SREBP-1C) and liver X receptor α (LXR α), and the transcriptional level was intimately related to dietary lipid addition (Matsuzaka et al., 2002; Kumadaki et al., 2008; Ducheix et al., 2011; Sun et al., 2013; Bae et al., 2016). Likewise, both *in vivo* and *in vitro* demonstrated that dietary fatty acids could markedly affect the *elovl6* expression through regulating related transcription factors for *L. crocea* and *O. mykiss* (Li et al., 2019; 2020). Besides, the *elovl6* plays a vital role in keeping fatty acids and energy balances in dealing with cold stress (Tan et al., 2015; Chen et al., 2018). Studies have found that the transcriptional level of *elovl6* was significantly increased in brown adipose tissue under the cold stress, and *elovl6* ^−/−^ mice showed lower heat-producing capability in brown adipose tissue (Tan et al., 2015). Similar result was also observed in *M. anguillicaudatus*, which found that the *elovl6* expression could be induced by the cold stress for producing fatty acids to maintain proper membrane fluidity (Chen et al., 2018). In addition to temperature stress, aquatic animals also often need face salinity and starvation stress. In aquatic animals, supply of energy is crucial for coping with salinity and starvation stress, as well as maintenance of suitable cell membrane fluidity is also important adaptive way during osmoregulation. However, to the best our knowledge, the roles of *elovl6* in the face of salinity and starvation stress are still poorly understood.

The mud crab, *Scylla paramamosain*, is a kind of important marine crustacean species (Ye et al., 2010). Because of high nutritional value, unique flavor, high output and high economic value, the mud crab has been widely cultured in the coastal areas of southern China with a yield of around 152,065 tons in 2021 (China Fishery Statistical Yearbook, 2022). The present study aimed to determine the molecular features of three *elovl6* and their expression profiles in reaction to ambient salinity, dietary fatty acids and starvation. These results may be beneficial for further understanding the functions of *elovl6* in fatty acid synthesis and regulatory mechanism in crustaceans.

## 2 Materials and methods

### 2.1 Nutrition experiment

Six isonitrogenous (45% crude protein) and isolipidic (9.5% crude lipid) experimental diets were prepared by substituting fish oil with 0% (FO group), 20% (SO-20 group), 40% (SO-40 group), 60% (SO-60 group), 80% (SO-80 group) and 100% (SO-100 group) soybean oil. The dietary protein sources were provided with casein and white fishmeal, and lipid sources were supplied by soybean oil, cholesterol, phospholipids and fish oil. The specific feed formula, diet making process, experimental design, experimental condition and sample collection have been described in our previous studies (Lin et al., 2017; Lin et al., 2018).

### 2.2 Salinity stress experiment

The 21-day salinity stress experiment was conducted in culture system of Ningde Normal University. The salinity was set as 27‰, 22‰, 17‰, 12‰ and 7‰. The crabs used in the present study were bought from a local crab farm in Sandu bay (Ningde, Fujian, China), and the crabs were temporarily cultured for adapting to the experimental environment and diets. Subsequently, ninety healthy crabs (initial average weight: 62.90 ± 1.98 g) with intact limbs were assigned to fifteen polypropylene buckets (Zhongkehai, Qingdao, China). There were five groups, each with three replicates, and each replicate with six crabs. The crabs were fed commercial diets twice daily (8:30 and 18:00) to apparent satiation during experiment. Feces and residual diets were removed once a day. During the salinity stress experiment, water quality parameters were as follows: the temperature ranged from 19.1°C to 22.4°C, oxygen concentration more than 5.0 mg L^−1^ and ammonia nitrogen lower than 0.05 mg L^−1^. At the end of the trial, the crabs were dissected to obtain the hepatopancreas and muscle samples after being starved for 24 h. Then, the samples were immediately frozen in liquid nitrogen and stored at −80°C for further treatment.

### 2.3 Starvation stress experiment

The crabs (64.51 ± 0.83 g) used in the present study were purchased from Sandu bay (Ningde, Fujian, China), and 4-week starvation stress experiment was performed in culture system of Ningde Normal University. The starvation stress experiment contained two treatments: starvation group (SG) and feeding group (FG). Thirty-six vigorous crabs were randomly divided into six polypropylene buckets after being temporarily reared for acclimatization. Each treatment has three replicates, and each replicate has six crabs. During the starvation stress experiment, the crabs in feeding group was fed twice daily (8:30 and 18:00) to apparent satiation with a local bivalve mollusc (*Sinonovacula constrzcta*), and starvation group was not fed any food. Uneaten feeds and feces were cleared once daily, and 30% water from each tank was exchanged per day. During the experiment, dissolved oxygen of water was more than 7.0 mg L^−1^, water temperature ranged from 15.3°C to 20.3°C and ammonia nitrogen was lower than 0.05 mg L^−1^. The hepatopancreas and muscle samples were collected and immediately frozen in liquid nitrogen after the experiment completed. Subsequently, the samples above were stored at −80°C for further analysis.

### 2.4 RNA isolation, first-strand cDNA synthesis and full-length cDNA cloning

Total RNA was extracted from the fresh hepatopancreas using Trizol reagent (Invitrogen, United States) according to the manufacturer’s instructions. After determining the quality and concentration of total RNA, SMARTer™ RACE cDNA Amplification kit (Clontech, United States) was used to produce the first-strand cDNA based on specification. The cDNA samples were kept in −20°C as subsequent cloning templates.

Three partial cDNA sequences of *elovl6s*, named *elovl6a*, *elovl6b* and *elovl6c*, were obtained from our previous transcriptome sequencing. Related primers were designed according to the sequences above, and the primers have been shown in Table 1. 5′ and 3′ rapid amplification of cDNA ends (RACE) methods were applied to clone the 5′ untranslated region (UTR) and 3′ UTR of three *elovl6s* using touch-down PCR (first round PCR) and nested PCR (second round PCR) strategies. The primers of elovl6a 3-1, elovl6a 5-1, elovl6b 3-1, elovl6b 5-1, elovl6c 3-1 and elovl6c 5-1 were applied to touch-down PCR, and the primers of elovl6a 3-2, elovl6a 5-2, elovl6b 3-2, elovl6b 5-2, elovl6c 3-2 and elovl6c 5-2 were used to nested PCR. The amplification program and reaction system of touch-down PCR and nested PCR have been shown in our previous studies (Lin et al., 2017; Lin et al., 2018). Target band was purified with SanPrep Column DNA Gel Extraction Kit (Sangon Biotech, Shanghai, China.), and then cloned into pMD 19-T simple vector (Takara, Dalian, China). The sequence information of positive clones was determined by sequencing with a commercial company (BGI, Shenzhen, China).

**TABLE 1:** Names and sequences of primers used in the present study.

Primer	Sequence (5′-3′)	Objective
Oligo-	AAG​CAG​TGG​TAT​CAA​CGC​AGA​GTACXXXXX	First-Strand cDNA Synthesis
UPM (long)	CTA​ATA​CGA​CTC​ACT​ATA​GGG​CAA​GCA​GTG​GTA​TCA​ACG​CAG​AGT	RACE-PCR
UPM (short)	CTA​ATA​CGA​CTC​ACT​ATA​GGG​C	RACE-PCR
NUP	AAGCAGTGGTATCAACGCAGAGT	RACE-PCR
M13F	CGC​CAG​GGT​TTT​CCC​AGT​CAC​GAC	PCR screening
M13R	AGC​GGA​TAA​CAA​TTT​CAC​ACA​GGA	PCR screening
β-actin R	GCG​GCA​GTG​GTC​ATC​TCC​T	qRT-PCR
Srebp-1 F	GCT​TCA​AGG​GAT​GAG​GTT​TGC	qRT-PCR
Srebp-1 R	GGA​TCT​TCT​GAG​GTC​CTG​AGG​TAC​T	qRT-PCR
β-actin F	GCC​CTT​CCT​CAC​GCT​ATC​CT	qRT-PCR
For elovl6a clone and qRT-PCR		
elovl6a 3–1	AGT​ACC​GCC​CTC​GAT​TTG​AGC​TCC​G	3′RACE
elovl6a 3–2	GGA​CCC​AGT​TTC​CTT​GAC​AAC​CGT​GT	3′RACE
elovl6a 5–1	CCA​CCC​ACA​CGG​TTG​TCA​AGG​AAA​CT	5′RACE
elovl6a 5–2	TCG​AGG​GCG​GTA​CTG​CAT​GTA​AAG​TTG	5′RACE
Q-elovl6a F	TCA​CTC​CTC​AAA​AAA​CCA​CGC	qRT-PCR
Q-elovl6a R	GCT​GAC​ACA​CGA​CAC​GCT​CAA	qRT-PCR
For elovl6b clone and qRT-PCR		
elovl6b 3–1	CGT​TAC​CTC​CAC​CAA​CTT​CAC​CTA​CCG	3′RACE
elovl6b 3–2	GCC​TTA​CGC​ACC​ACG​CCT​GAA​ATG	3′RACE
elovl6b 5–1	CAG​CAT​TTC​AGG​CGT​GGT​GCG​TAA​G	5′RACE
elovl6b 5–2	TCG​GCG​GGA​GTG​TCA​GGT​CGT​AG	5′RACE
Q-elovl6b F	TTC​ACC​TAC​CGC​TAC​ACC​TTC​A	qRT-PCR
Q-elovl6b R	ACT​TGG​GTC​GCT​TTT​CCA​TCA​C	qRT-PCR
For elovl6c clone and qRT-PCR		
elovl6c 3–1	CTA​CAT​GGT​GGG​CGC​CTA​CAT​GGC	3′RACE
elovl6c 3–2	TGT​TCA​CGT​TGA​GCA​AGG​TGC​CAG​A	3′RACE
elovl6c 5–1	TGG​CAC​CTT​GCT​CAA​CGT​GAA​CAT​C	5′RACE
elovl6c 5–2	GGT​CAA​AAG​CAG​GTC​GGG​TCT​CCA	5′RACE
Q-elovl6c F	TCT​ACG​GCG​GAA​ACT​GGG​TG	qRT-PCR
Q-elovl6c R	TGC​TTG​CGG​AGG​TCA​AAA​GC	qRT-PCR

X, undisclosed base in the proprietary SMARTer, oligo sequence.

### 2.5 Sequence and phylogenetic analysis

Homology searches were performed with BLAST at the National Center for Biotechnology Information (http://www.ncbi.nlm.nih.gov/). The multiple alignments were created using the DNAMAN software. Transmembrane structure was predicted utilizing the TMHMM 2.0 (https://services.healthtech.dtu.dk/services/TMHMM-2.0/). The phylogenetic analysis based on the amino acid sequences was constructed by the Neighbour-Joining algorithm with the software MEGA version 7.0.

### 2.6 Quantitative real-time PCR

Six mud crabs (average weight: 103.60 ± 6.20 g) were used to investigate the *elovl6a, elovl6b* and *elovl6c* expression levels in different tissues (epidermis, gill, hepatopancreas, cranial ganglia, eyestalk, thoracic ganglia, stomach, intestine, muscle and heart) by using quantitative real-time PCR. Likewise, quantitative real-time PCR was also applied to detect the *elovl6a*, *elovl6b* and *elovl6c* mRNA levels in response to dietary fatty acids, salinity stress and starvation stress. The *β-actin* gene from *S. paramamosain* was selected as reference for internal standardization. Primers used in quantitative real-time PCR were given in Table 1. The amplification program and reaction system of quantitative real-time PCR have been described in our previous studies used for determining tissue distribution as well as *elovl6a*, *elovl6b* and *elovl6c* expression levels in response to dietary fatty acids (Lin et al., 2017; Lin et al., 2018). In addition, as for salinity and starvation stress experiment, the total RNA of muscle and hepatopancreas was isolated by using TRNzol universal Reagent (Tiangen, Beijing, China). The single-strand cDNA was synthesized using PrimeScript^®^ RT reagent Kit with gDNA Eraser with 1 μg of total RNA, and the obtained cDNA was diluted by 4 times using ultra-pure water for further analysis. Five different thinned cDNA samples were used to determine the standard curves. The amplification efficiency of primers used in the present study was between 95% and 105% by counting with formula *E* = 10^(−1/Slope)^—1. Quantitative real-time PCR was performed in a total volume of 20 μL including 10 μL 2 × ChamQ universal SYBR qPCR Master Mix (Q711–02/03, Vazyme Biotech Co., Ltd., Nanjing, China), 1.0 μL of the diluted cDNA template, 0.4 μL of each primer (10 μM) and 8.2 μL of sterile distilled H_2_O. The program of quantitative real-time PCR was 95°C for 30 s, followed by 40 cycles of 95°C for 10 s and 60°C for 30 s, and then a dissociation curve (95°C for 15 s, 60°C for 60 s and 95°C for 15 s) was performed to identify unicity of PCR product. The relative mRNA expression levels were calculated by 2^−ΔΔCt^ method (Livak and Schmittgen, 2001).

### 2.7 Statistical analysis

Results were shown in the form of means ± SEM (standard error of the mean). After checking homogeneity and normality, one-way analysis of variance (ANOVA) followed by Duncan’s multiple comparison test was used to determine the differences of tissue distribution, nutrition experiment and salinity stress experiment. In addition, independent-samples *t*-test was applied to analyze differences in starvation stress experiment. All statistical analysis was carried out by SPSS 20.0 (SPSS, Chicago, IL, United States), and *p* values less than 0.05 was considered to be statistically significant.

## 3 Results

### 3.1 cDNA cloning and sequence analysis

**FIGURE 1:**
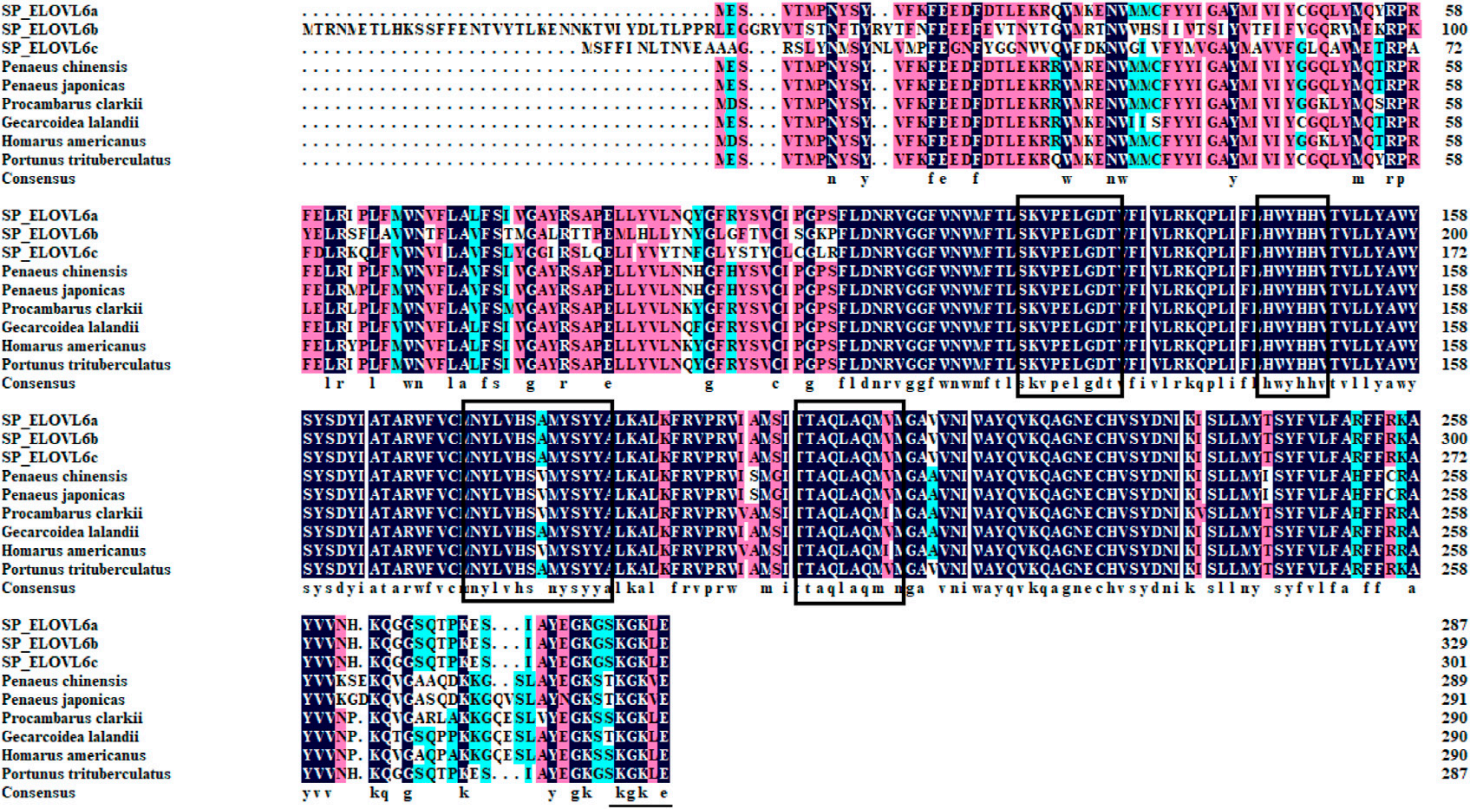
Multiple alignments of the ELOVL6 amino acid sequences between *Scylla paramamosain* and other crustaceans. The threshold for similarity shading was set at 50%. Identical residues are shaded black. Amino acid residues that are conserved in at least 75% and 50% of sequences are shaded in pink and cyan, respectively. The motifs highly conserved are boxed among elongases, and the endoplasmic reticulum retention signal is underlined. SP_ELOVL6a: *Scylla paramamosain* ELOVL6a; SP_ELOVL6b: *Scylla paramamosain* ELOVL6b; SP_ELOVL6c: *Scylla paramamosain* ELOVL6c. *Procambarus clarkii* (XP_045621678), *Homarus americanus* (XP_042209792), *Penaeus japonicas* (XP_042885566), *Portunus trituberculatus* (XP_045136269), *Penaeus chinensis* (XP_047487575) and *Gecarcoidea lalandii* (QKG32709).

The full-length cDNA sequences of *elovl6a*, *elovl6b* and *elovl6c* were obtained by overlapping the corresponding expressed sequence tags (ESTs) with the amplified fragments using the RACE technology. The sequences of *elovl6a*, *elovl6b* and *elovl6c* were submitted to GenBank getting the accession numbers MF784574, OQ863017 and OQ863018 respectively. The complete sequence of *elovl6a* was 1345 bp containing a 5′-UTR of 176 bp, a 3′-UTR of 305 bp with a poly A tail and an open reading frame (ORF) of 864 bp encoding a putative protein of 287 amino acids, and the full-length sequence of *elovl6b* was 1419 bp and consists of a 990 bp ORF from 126 bp to 1115 bp encoding a putative protein of 329 amino acids, 125 bp of 5′-UTR and 304 bp of 3′-UTR with a poly A tail. In addition, the obtained *elovl6c* sequence was 1375 bp in full length with 167 bp of 5′-UTR and 302 bp of 3′-UTR including poly A tail, which contained an ORF of 906 bp encoding a putative protein of 301 amino acids. All the ELOVL6s possessed the endoplasmic reticulum retention signal (KXKXX) and membrane-spanning domains (Supplemented Fig. s1-3). Multiple alignments of ELOVL6a, ELOVL6b and ELOVL6c indicated that the predicted amino acid sequences contained characteristic conserved motifs of the microsomal ELOVL family, like HXXHH (histidine box), KXXEXXDT, NXXXHXXMYXYY and TXXQXXQ (Figure 1).

### 3.2 Homology and phylogenetic analysis

**FIGURE 2:**
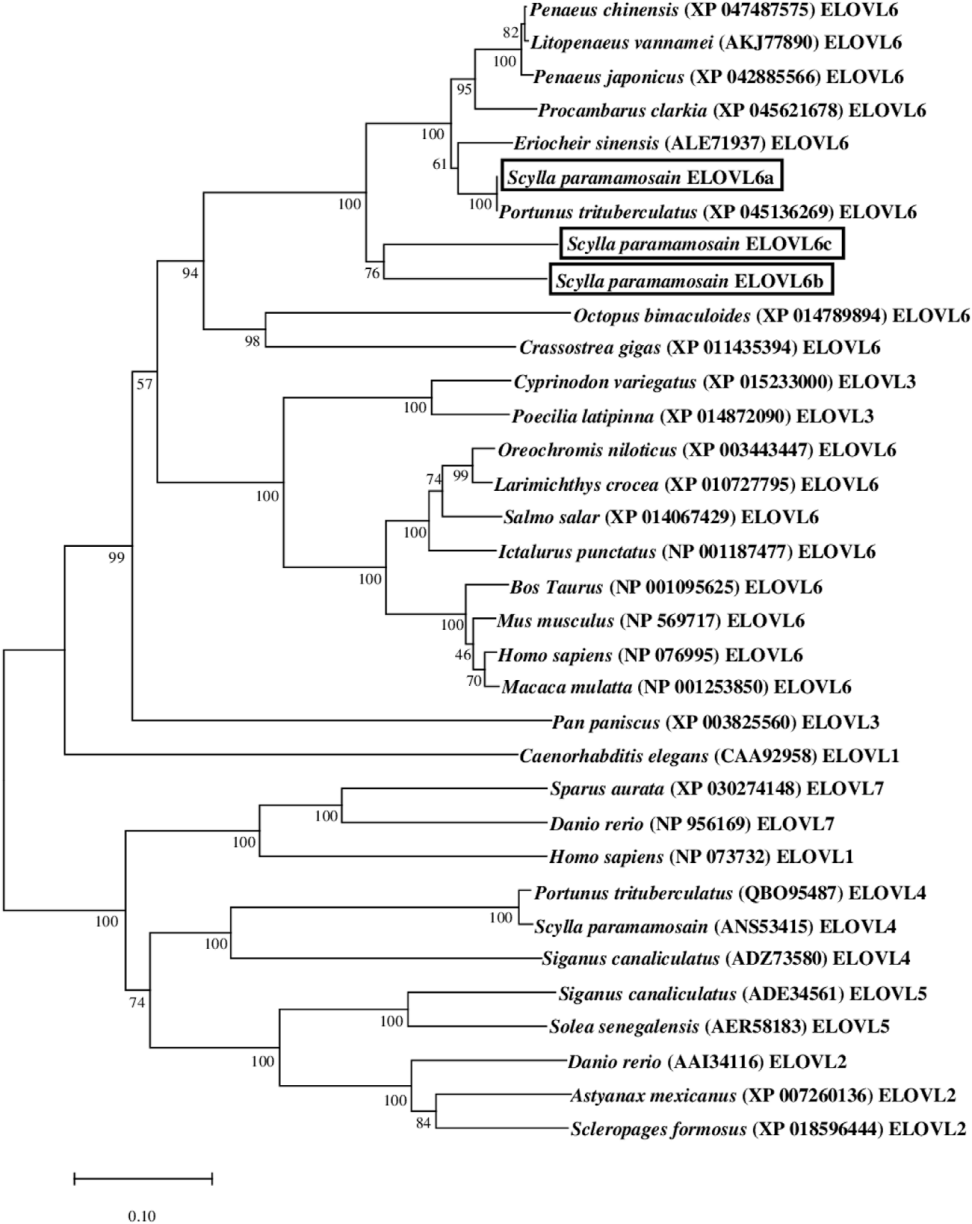
Phylogenetic analysis between the amino acid sequences of *Scylla paramamosain* ELOVL6 and 31 available ELOVL sequences. The tree was constructed using the neighbor joining method with MEGA 7.0. The horizontal branch length is proportional to amino acid substitution rate per site. Numbers represent the frequencies with which the tree topology presented was replicated after 1000 bootstrap iterations.

The results of phylogenetic tree showed that the three mud crab ELOVL6 gathered together with their corresponding orthologues, and separated with the ELOVL1, ELOVL2, ELOVL3, ELOVL4, ELOVL5 and ELOVL7. The mud crab ELOVL6a clustered most closely to ELOVL6 from *Portunus trituberculatus*, and further clustered with *E. sinensis*. In addition, the mud crab ELOVL6b and ELOVL6c gathered alone into a single branch and then clustered with other ELOVL6 from crustaceans (Figure 2).

### 3.3 Tissue distribution

**FIGURE 3:**
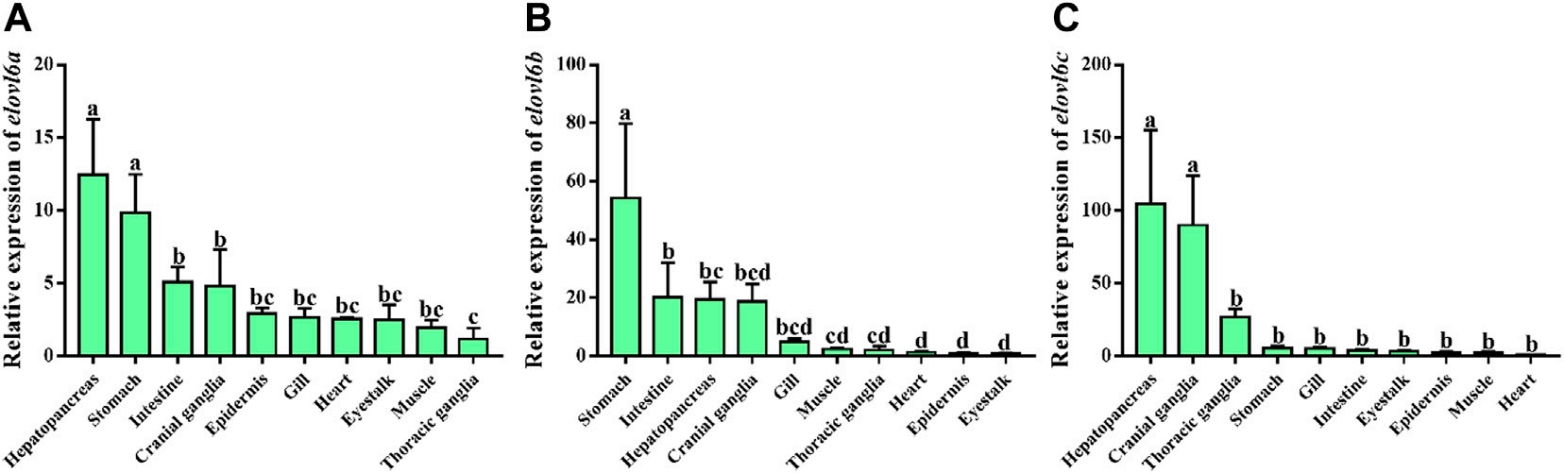
Relative mRNA levels of *elovl6* in different tissues of *Scylla paramamosain*. Vertical bars represented mean ± SEM (*n* = 6) for each tissue. Letters show significant differences (*p* < 0.05) among tissues as determined by one-way ANOVA followed by Duncan’s multiple comparison test.

Quantitative real-time PCR was used to analyze mRNA levels of *elovl6a*, *elovl6b* and *elovl6c* in the tissues of healthy crabs, including thoracic ganglia, stomach, heart, gill, epidermis, hepatopancreas, intestine, muscle, eyestalk and cranial ganglia. As illustrated in Figure 3, *elovl6a*, *elovl6b* and *elovl6c* could be detected in all the examined tissues, but existed obvious differences in expression levels. The relatively abundant expression of *elovl6a* was observed in hepatopancreas and stomach, moderate expression in intestine and cranial ganglia and low expression in epidermis, gill, heart, eyestalk, muscle and thoracic ganglia. The *elovl6c* was expressed at significantly higher levels in hepatopancreas and stomach compared to other tissues (*p* < 0.05). By contrast, the *elovl6b* was mainly expressed in the stomach, followed by the intestine and hepatopancreas.

### 3.4 Transcriptional levels of *elovl6a*, *elovl6b* and *elovl6c* in response to dietary fatty acids

**FIGURE 4:**
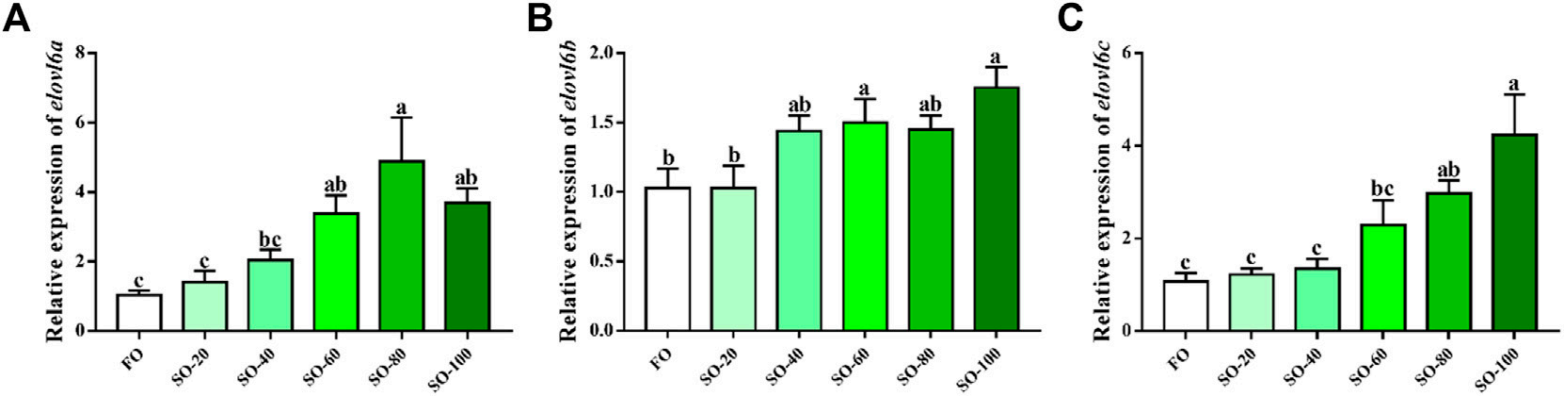
Relative expression levels of *elovl6* in hepatopancreas of *Scylla paramamosain* fed six experimental diets. Bars with different superscripts are significantly different (*p* < 0.05, one-way ANOVA and Duncan’s multiple comparison test). FO means that fish oil is the only dietary lipid source, numerical values after SO refer to the percentage of dietary fish oil replaced by soybean oil.

The mRNA levels of *elovl6s* in hepatopancreas were observably influenced by the dietary fatty acids (*p* < 0.05). The crabs fed SO-60, SO-80 and SO-100 diets showed markedly higher *elovl6a* mRNA levels than those fed FO and SO-20 diets (*p* < 0.05). There was no significant difference between the FO and SO-20 groups in the *elovl6a* expression (*p* > 0.05). The mRNA levels of *elovl6b* in the SO-60 and SO-100 groups were dramatically upregulated when compared with the FO group. (*p* < 0.05). Although no significant differences were detected among the FO, SO-40 and SO-80 groups, the SO-40 and SO-80 groups had the higher *elovl6b* mRNA levels than the FO group (*p* > 0.05). In addition, the *elovl6c* transcriptional levels showed increasing tendency with the increased replacement of dietary fish oil by soybean oil, and the SO-80 and SO-100 groups exhibited significantly higher *elovl6c* transcriptional levels than the FO group (*p* < 0.05) (Figure 4).

### 3.5 Transcriptional levels of *elovl6a*, *elovl6b*, *elovl6c* and *srebp-1* in response to ambient salinity

**FIGURE 5:**
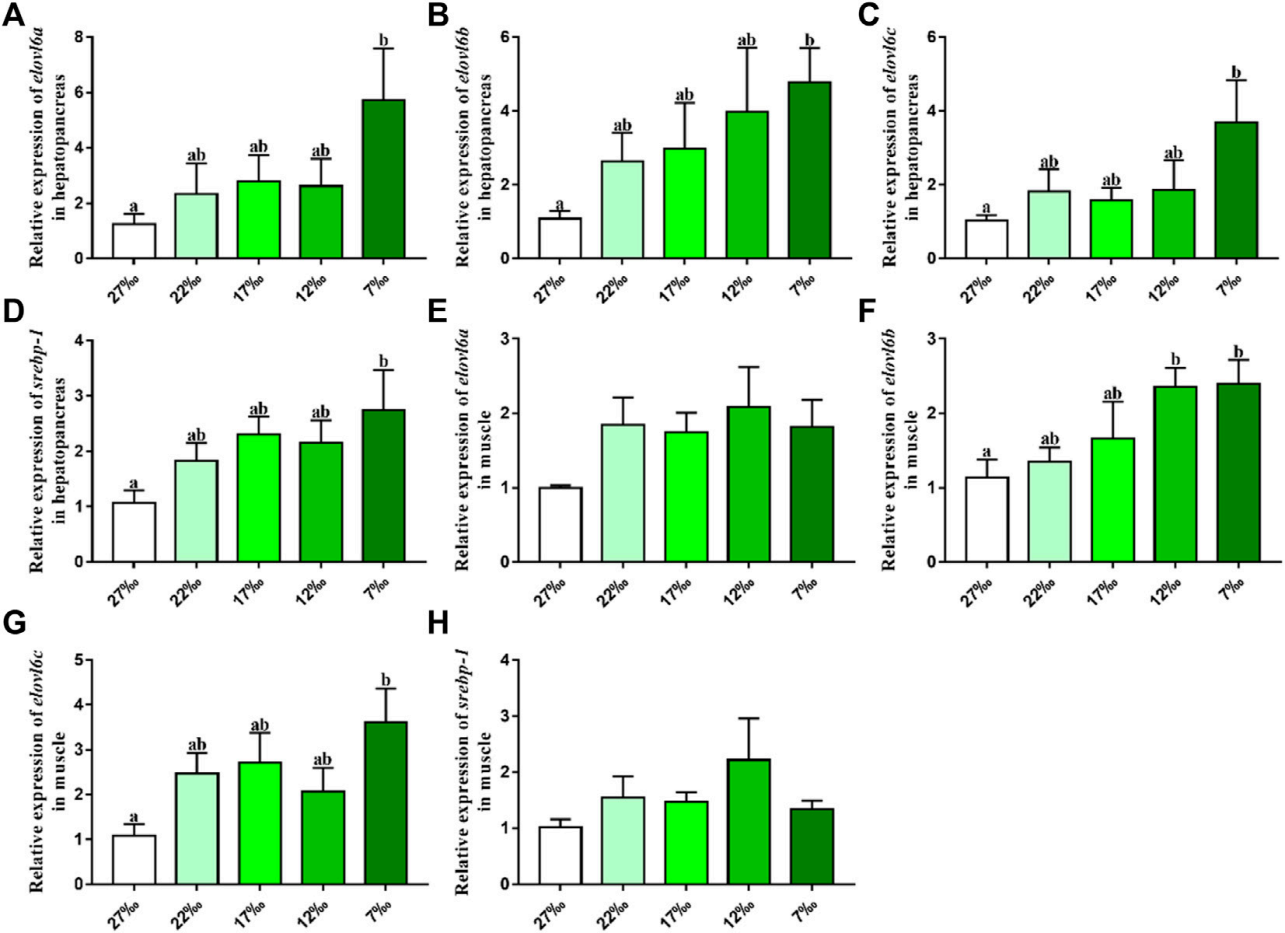
Relative expression levels of *elovl6* and *srebp-1* in hepatopancreas and muscle of *Scylla paramamosain* in response to salinity stress. Bars with different superscripts are significantly different (*p* < 0.05, one-way ANOVA and Duncan’s multiple comparison test). Numerical values refer to seawater salinity.

The effects of ambient salinity on the mRNA levels of *elovl6a*, *elovl6b*, *elovl6c* and *srebp-1* in hepatopancreas and muscle are presented in Figure 5. Compared with the 27‰ salinity group, the mRNA levels of *elovl6a*, *elovl6b*, *elovl6c* and *srebp-1* in hepatopancreas were upregulated in the 22‰, 17‰, 12‰ and 7‰ salinity groups, and the 7‰ salinity group showed a significant difference with the 27‰ salinity group (*p* < 0.05). There were no significant differences in *elovl6a* and *srebp-1* expression of muscle, although the 22‰, 17‰, 12‰ and 7‰ salinity groups had the higher levels than the 27‰ salinity group (*p* > 0.05). The *elovl6b* and *elovl6c* transcriptional levels in the 27‰ salinity group were also lower than the 22‰, 17‰, 12‰ and 7‰ salinity groups. In addition, the *elovl6c* mRNA levels in 7‰ salinity group and *elovl6b* transcriptional levels in 7‰ and 12‰ salinity groups exhibited markedly higher values than the 27‰ salinity group (*p* < 0.05).

### 3.6 Transcriptional levels of *elovl6a*, *elovl6b*, *elovl6c* and *srebp-1* in response to starvation stress

**FIGURE 6:**
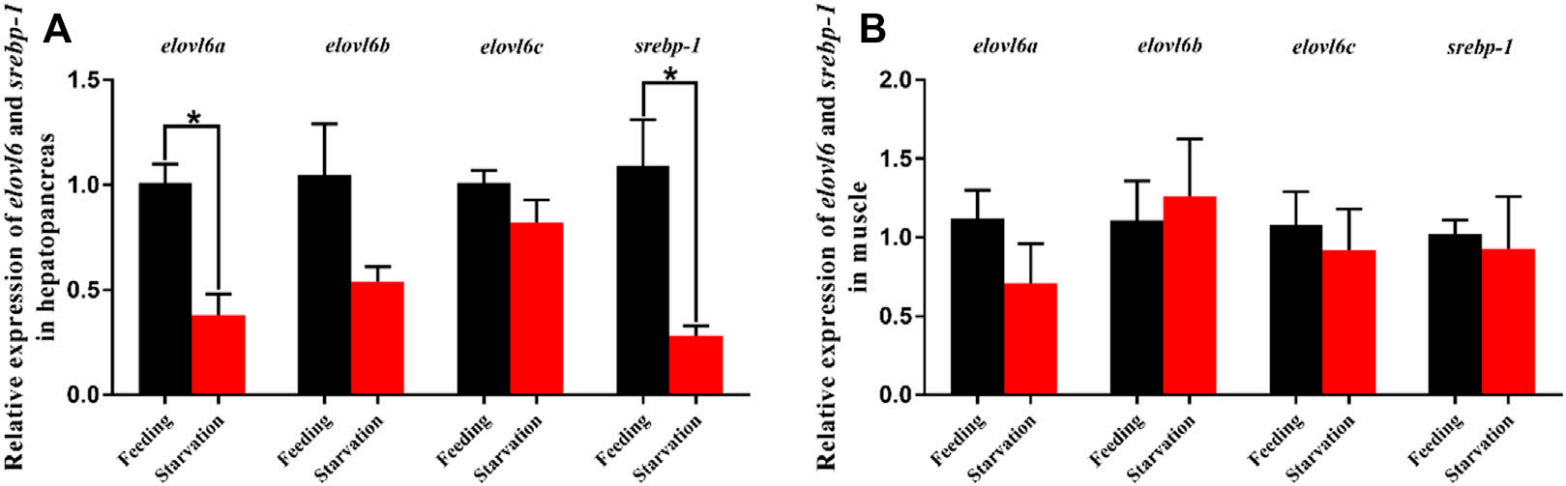
Relative expression levels of *elovl6* and *srebp-1* in hepatopancreas and muscle of *Scylla paramamosain* in response to starvation stress. * indicates significant difference between feeding group and starvation group (*p* < 0.05, independent-samples *t*-test).

The effects of starvation stress on the mRNA levels of *elovl6a*, *elovl6b*, *elovl6c* and *srebp-1* in hepatopancreas and muscle are shown in Figure 6. Compared with the feeding group, the *elovl6a*, *elovl6b*, *elovl6c* and *srebp-1* transcriptional levels in hepatopancreas were downregulated in the starvation group. The *elovl6a* and *srebp-1* expression levels in hepatopancreas of starvation group were dramatically lower than the feeding group (*p* < 0.01). Additionally, no significant differences in *elovl6a*, *elovl6b*, *elovl6c* and *srebp-1* expression levels of muscle were observed between the starvation group and feeding group (*p* > 0.05).

## 4 Discussion

As a final elongase participated in LCFAs *de novo* in conjunction with fatty acid synthase and stearoyl-CoA desaturase, the ELOVL6 was located in endoplasmic reticulum, which possessed the ability to elongate C16:1n-7 and C16:0 to C18:1n-7 and C18:0 respectively (Green et al., 2010; Shi et al., 2017). Previous studies have exhibited that the ELOVL6 was closely related to metabolic diseases and energy balance in mammal and fish (Takashi et al., 2012; Motoko et al., 2015; Tan et al., 2015; Zhao et al., 2017; Chen et al., 2018; Nakamura et al., 2018; Su et al., 2018), while few studies were reported in crustaceans. In the present study, three *elovl6* isoforms were isolated from the *S. paramamosain*, and the deduced amino acids have the typical structural features of ELOVL protein family members (Tocher, 2015), such as conserved motifs (KXXEXXDT, NXXXHXXMYXYY and TXXQXXQ), histidine box (HXXHH) and transmembrane regions. The phylogenetic analysis showed that the *S. paramamosain* ELOVL6 gathered together with their orthologues from crustaceans and separated with the ELOVL1, ELOVL2, ELOVL3, ELOVL4, ELOVL5 and ELOVL7, which further supported that the isolated genes were *elovl6*. The ELOVL6a from *S. paramamosain* clustered most closely to ELOVL6 from *P. trituberculatus* and *E. sinensis*, which indicated that they have an intimate relationship. In addition, the ELOVL6b and ELOVL6c from *S. paramamosain* gathered alone into a single branch, suggesting ELOVL6b and ELOVL6c have a closer genetic relationship than ELOVL6a and ELOVL6 from other crustaceans.

Studies in mice have indicated that the *elovl6* was mainly expressed in liver and brain (Moon et al., 2001; Matsuzaka et al., 2002; Bae et al., 2016). Similar results were also observed in *L. crocea* and *O. mykiss*, which found that the high expression of *elovl6* was detected in the liver, brain and eye (Li et al., 2019; Li et al., 2020). By contrast, three *elovl6* isoforms from *M. anguillicaudatus* exhibited different expression patterns, and muscle and ovary were the main expression sites (Chen et al., 2018). The results of present study showed that the highest expression levels of the *elovl6a* and *elovl6c* from *S. paramamosain* were the hepatopancreas. This result was consistent with a past study of Shi et al. (2016), who detected that *elovl6* was highly expressed in hepatopancreas of *E. sinensis*. Normally, the hepatopancreas is considered as a main lipid metabolism and storage organ akin to liver of vertebrates (Wen et al., 2001; Tian et al., 2023). The results above may indicate that the *elovl6a* and *elovl6c* mainly acted in the hepatopancreas in *S. paramamosain*. In addition, digestive organs are now regarded as an important site of fatty acid metabolism, at least in salmonids (Bell et al., 2003). The present study also found that the relatively abundant expression of *elovl6b* was observed in intestine and stomach, and the *elovl6a* also had high expression levels, suggesting *elovl6a* and *elovl6b* may plays an important role in fatty acid synthesis of these tissues in *S. paramamosain*.

Previous studies have demonstrated that the expression of *elovl6* could markedly affect by dietary fatty acids (Matsuzaka et al., 2002; Leroux et al., 2016; Shi et al., 2016; Li et al., 2019; Li et al., 2020). In mice, compared with fat-free diet, the diets added with eicosapentaenoic acid or linoleates significantly suppressed the *elovl6* expression, and the reduction was more obvious in fish oil rich in docosahexaenoic acid and eicosapentaenoic acid (Matsuzaka et al., 2002). In addition, studies found that *O. mykiss* fed diets containing fish oil exhibited higher the transcriptional level of *elovl6* in liver than those fed diets with soybean oil or linseed oil (Li et al., 2019). On the contrary, results from *L. crocea* have exhibited that the mRNA levels of *elovl6* in liver were observably upregulated in the soybean oil, linseed oil, or palm oil groups when compared with the fish oil group. Besides, hepatocytes from *L. crocea* treated with linoleic acid, α-linolenic acid or palmitic acid also obtained similar results above, and this increase may be regulated by related transcription factors like hepatocyte nuclear factor 1α (HNF1α) and retinoid X receptor α (RXRα) (Li et al., 2020). Likewise, the present study also found that replacing fish oil with soybean oil could significantly increase the transcriptional levels of three *elovl6* in hepatopancreas of *S. paramamosain*. This result was consistent with a study from *E. sinensis*, which observed that soybean oil group had markedly higher expression of *elovl6* than the fish oil group (Shi et al., 2016). Our past study has detected that compared with fish oil, soybean oil markedly upregulated the *srebp-1* expression, and the SREBP-1, as a transcription factor, can activate target gene expression like *elovl6* (Hao et al., 2018). Thus, we speculated that soybean oil rich in linoleic acid promoted the *elovl6* expression possibly through activating *srebp-1* expression in the present study. Additionally, the expression of *elovl6a* and *elovl6c* in hepatopancreas were more sensitive to dietary fatty acids than the *elovl6b* probably because these two genes are mainly expressed in hepatopancreas.

Besides, the expression of *elovl6* could also markedly affect by the environmental factors. Previous studies have proved that the *elovl6* plays a crucial role in regulating energy expenditure and fatty acid metabolism in adaptation to cold stress (Tan et al., 2015; Chen et al., 2018). In mice, the transcriptional level of *elovl6* was markedly upregulated in brown adipose tissue under the cold stress, and *elovl6* ^−/−^ mice exhibited lower heat-producing capability in brown adipose tissue (Tan et al., 2015). Consistently, Chen et al. (2018) found that the expression of three *elovl6* isoforms from *M. anguillicaudatus* could be induced by the cold stress for keeping energy balances and producing fatty acids to maintain proper membrane fluidity. In addition to temperature stress, aquatic animals often require cope with stresses of salinity changes and food scarcity. To the best our knowledge, the present study was the first time to investigate the *elovl6* expression in respond to salinity and starvation stress. The results showed that compared with the normal sea water group (27‰), the expression of *srebp-1*, *elovl6a*, *elovl6b* and *elovl6c* were upregulated in the low salinity groups, particularly in 7‰, suggesting that three *elovl6* may play a crucial role in salinity adaptation for *S. paramamosain*. The possible reason for this result above was that the expression of transcription factors, SREBP-1, could be activated by low salinity, which further promoted the expression of downstream target gene (*elovl6*) for synthesizing suitable fatty acids to maintain membrane fluidity. In addition, the starvation group exhibited lower expression of *srebp-1*, *elovl6a*, *elovl6b* and *elovl6c* than the feeding group. It could be speculated that more fatty acids are preferentially used for providing energy rather than synthesis when food is scarce, therefore three *elovl6* showed lower expression levels. Furthermore, the hepatopancreas was more sensitive to starvation stress than the muscle, and this was related to the hepatopancreas as a center of lipid metabolism.

In conclusion, the three *elovl6* cDNA sequences of *S. paramamosain* were isolated in the present study, and the deduced amino acids exhibited the typical structural features of ELOVL protein family members. The results of tissue distribution indicated that the *elovl6a* and *elovl6c* highly expressed in the hepatopancreas, while the relatively abundant expression of *elovl6b* was observed in intestine and stomach. In addition, the dietary fatty acids and ambient salinity significantly increases the transcriptional levels of three *elovl6*, and starvation stress could inhibit three *elovl6* expression. These results may contribute to understand functions of *elovl6* in fatty acid synthesis and regulatory mechanisms in crustaceans.

## Data Availability

The datasets presented in this study can be found in online repositories. The sequences of elovl6a, elovl6b and elovl6c were submitted to GenBank (https://www.ncbi.nlm.nih.gov/) with the accession numbers MF784574, OQ863017 and OQ863018, respectively.
