# Supplementary material for: Cloning and expression characterization of elongation of very long-chain fatty acids protein 6 (elovl6) with dietary fatty acids, ambient salinity and starvation stress in Scylla paramamosain
Source: Front Physiol. 2023 Jul 12;14:1221205. doi: 10.3389/fphys.2023.1221205 (PMC10382226; doi:10.3389/fphys.2023.1221205)
Supplement: Supplementary file 1 [file Table1.DOCX]

1 GCCCGCTCACTCCTCAAAAAACCACGCGTTTCCTTTCCGCTACCACCAGTCGTCACCTCCCAGCCCTTCATTCTGCATCAGTCCGT

87 TGCTTGAGCGTGTCGTGTGTCAGCCGGTCTGTGTGTCGCTGTTGACCAAGTGAAACCCAAGCAACCCCAGCCGCTACCAAGAAACCAACC

177 **ATG**GAGTCGGTCACCATGCCAAATTATTCCTATGTCTTCAAGTTCGAGGAGGACTTTGACACCTTGGAAAAGCGACAATGGATGAAAGAG

1 M E S V T M P N Y S Y V F K F E E D F D T L E K R Q W M K E

267 AACTGGATGATGTGCTTTTACTACATCGGCGCCTACATGATTGTAATATACTGCGGCCAACTTTACATGCAGTACCGCCCTCGATTTGAG

31 N W M M C F Y Y I G A Y M I V I Y C G Q L Y M Q Y R P R F E

357 CTCCGCATTCCTCTCTTCATGTGGAACGTGTTCCTGGCGCTTTTTTCCATCTGGGGAGCTTACCGAAGTGCTCCTGAATTACTCTACGTC

61 L R I P L F M W N V F L A L F S I W G A Y R S A P E L L Y V

447 CTCAACCAATACGGCTTTAGATACTCCGTCTGCATCCCGGGACCCAGTTTCCTTGACAACCGTGTGGGTGGGTTCTGGAACTGGATGTTC

91 L N Q Y G F R Y S V C I P G P S F L D N R V G G F W N W M F

537 ACGTTGAGCAAGGTGCCAGAGCTCGGCGACACAGTGTTCATTGTGCTAAGGAAGCAGCCACTAATCTTCCTCCACTGGTACCACCACGTC

121 T L S K V P E L G D T V F I V L R K Q P L I F L H W Y H H V

627 ACTGTCCTCCTCTATGCCTGGTACTCCTACTCCGACTACATCGCCACCGCCCGTTGGTTTGTCTGCATGAACTACCTTGTCCACAGTGCC

151 T V L L Y A W Y S Y S D Y I A T A R W F V C M N Y L V H S A

717 ATGTACAGCTACTACGCCCTCAAGGCCCTCAAGTTCCGGGTTCCCCGCTGGATTGCCATGAGCATCACCACAGCTCAGTTGGCCCAGATG

181 M Y S Y Y A L K A L K F R V P R W I A M S I T T A Q L A Q M

807 GTGATGGGAGCAGTAGTAAACATTTGGGCCTACCAGGTGAAACAGGCTGGCAATGAGTGTCATGTCTCCTACGATAACATTAAAATCTCC

211 V M G A V V N I W A Y Q V K Q A G N E C H V S Y D N I K I S

897 CTCCTCATGTACACATCCTACTTTGTTCTCTTCGCTCGCTTCTTCCGCAAAGCTTATGTTGTGAACCACAAGCAAGGAGGCTCTCAGACA

241 L L M Y T S Y F V L F A R F F R K A Y V V N H K Q G G S Q T

987 CCCAAGGAGTCTATTGCTTATGAAGGGAAAGGTAGCAAGGGTAAACTGGAA**TAA**AAGCCTATCTTTACTGGGATTTATAGGGAATAAAAC

271 P K E S I A Y E G K G S K G K L E

1077 TCATTATTGGTCTGTCAAACACATGGGTCCCACTAATGGCCAATAGCTTGCTTGTATCTTACACATGTTTATTTACTACCAGCTGAGGCT

1167 ATTGCCATATCTAAATTTCACTGTAAAGTATTACAGTACAGTATATGTATAAGGAAATGGTATTTTACAGTTTATTCTTTCTTCAGTTTG

1257 TCAGTGAATTTTTAACATTTCATCAGTGGTTGCTGACAACAAGAATTAAGAATTTTGCAATAAAAAAAAAAAAAAAAAAAAAAAAAAAA

**Figure s1** Nucleotide and deduced amino acid sequences of *elovl6a*. The nucleotides and amino acids are numbered along the left margin respectively. The start (ATG) and stop (TAA) codons are marked in bold. Membrane-spanning domains are boxed, and endoplasmic reticulum retention signal is bold shaded.
