# Supplementary material for: Cloning and expression characterization of elongation of very long-chain fatty acids protein 6 (elovl6) with dietary fatty acids, ambient salinity and starvation stress in Scylla paramamosain
Source: Front Physiol. 2023 Jul 12;14:1221205. doi: 10.3389/fphys.2023.1221205 (PMC10382226; doi:10.3389/fphys.2023.1221205)
Supplement: Supplementary file 2 [file Table2.DOCX]

1 TCCTCCTCCTCCTCCTCCATACAGTTCTTCAATGA

36 TTCAGGATCCCGTGCAGTGAGCTGAGAACTAATACTGGACTGAGGACTTAGTTGCGTGATTCTGTGTACCGGAAAAGCTTATCTCTCGCC

126  **atg**acaaggaatatggagacactgcataaatccagcttctttgaaaacacggtgtacacgctgaaggagaacaacaaaacatggatctac

1 M T R N M E T L H K S S F F E N T V Y T L K E N N K T W I Y

216 gacctgacactcccgccgaggctggagggaggccgctacgttacctccaccaacttcacctaccgctacaccttcaacttcgaggaggag

31 D L T L P P R L E G G R Y V T S T N F T Y R Y T F N F E E E

306 tttgaggtgaccaactacaccgggtggatgagaaccaactggtggcacagcatcatctggacgagcatctacgtcacctttatcttcgtg

61 F E V T N Y T G W M R T N W W H S I I W T S I Y V T F I F V

396 ggtcagcgggtgatggaaaagcgacccaagtatgagctgcggagcttcctggctgtctggaacactttcttggccgtgttcagtaccatg

91 G Q R V M E K R P K Y E L R S F L A V W N T F L A V F S T M

486 ggcgccttacgcaccacgcctgaaatgctgcatctcctctacaactacggcctcggcttcaccgtctgcatatcgggaaaacctttcctt

121 G A L R T T P E M L H L L Y N Y G L G F T V C I S G K P F L

576 gacaaccgtgtgggtgggttctggaactggatgttcacgttgagcaaggtgccagagctcggcgacacagtgttcattgtgctaaggaag

151 D N R V G G F W N W M F T L S K V P E L G D T V F I V L R K

666 cagccactaatcttcctccactggtaccaccacgtcactgtcctcctctatgcctggtactcctactccgactacatcgccaccgcccgt

181 Q P L I F L H W Y H H V T V L L Y A W Y S Y S D Y I A T A R

756 tggtttgtctgcatgaactaccttgtccacagtgccatgtacagctactacgccctcaaggccctcaagttccgggttccccgctggatt

211 W F V C M N Y L V H S A M Y S Y Y A L K A L K F R V P R W I

846 gccatgagcatcaccacagctcagttggcccagatggtgatgggagcagtagtaaacatttgggcctaccaggtgaaacaggctggcaat

241 A M S I T T A Q L A Q M V M G A V V N I W A Y Q V K Q A G N

936 gagtgtcatgtctcctacgataacattaaaatctccctcctcatgtacacatcctactttgttctcttcgctcgcttcttccgcaaagct

271 E C H V S Y D N I K I S L L M Y T S Y F V L F A R F F R K A

1026 tatgttgtgaaccacaagcaaggaggctctcagacacccaaggagtctattgcttatgaagggaaaggtagcaagggtaaactggaa**taa**

301 Y V V N H K Q G G S Q T P K E S I A Y E G K G S K G K L E

1116 AAGCCTATCTTTACTGGGATTTATAGGGAATAAAACTCATTATTGGTCTGTCAAACACATGGGTCCCACTAATGGCCAATAGCTTGCTTG

1206 TATCTTACACATGTTTATTTACTACCAGCTGAGGCTATTGCCATATCTAAATTTCACTGTAAAGTATTACAGTACAGTATATGTATAAGG

1296 AAATGGTATTTTACAGTTTATTCTTTCTTCAGTTTGTCAGTGAATTTTTAACATTTCATCAGTGGTTGCTGACAACAAGAATTAAGAATT

1386 TTGCAAAAAAAAAAAAAAAAAAAAAAAAAAAAAA

**Figure s2** Nucleotide and deduced amino acid sequences of *elovl6b*. The nucleotides and amino acids are numbered along the left margin respectively. The start (ATG) and stop (TAA) codons are marked in bold. Membrane-spanning domains are boxed, and endoplasmic reticulum retention signal is bold shaded
