# Supplementary material for: Cloning and expression characterization of elongation of very long-chain fatty acids protein 6 (elovl6) with dietary fatty acids, ambient salinity and starvation stress in Scylla paramamosain
Source: Front Physiol. 2023 Jul 12;14:1221205. doi: 10.3389/fphys.2023.1221205 (PMC10382226; doi:10.3389/fphys.2023.1221205)
Supplement: Supplementary file 3 [file Table3.DOCX]

1 CTCCTGCCTCCGCGCCACCAACACCACCACTCTCCACCCGGCCGTCAGCTGTTCTCTGCCTTCCCTAGCCGTGGTGA

78 TTGCGTACGTGTTGGTGCCATAGACTCCTGGACCTGCGACGAGAAAGTGTACTGTTTGAAAATACGTGAAAAAAAAACTAACACGACAAA

168 **ATG**AGTTTCTTCATCAATTTGACGAACGTGGAGGCAGCTGCCGGCCGGTCCTTATACAACATGTCCTATAACCTGGTGATGCCCTTCGAG

1 M S F F I N L T N V E A A A G R S L Y N M S Y N L V M P F E

258 GGGAACTTCTACGGCGGAAACTGGGTGCAATGGTTCGACAAGAATTGGGGCATCGTATTCTACATGGTGGGCGCCTACATGGCCGTGGTG

31 G N F Y G G N W V Q W F D K N W G I V F Y M V G A Y M A V V

348 TTCGGCCTACAGGCATGGATGGAGACCCGACCTGCTTTTGACCTCCGCAAGCAGCTATTTGTGTGGAACGTTATCCTGGCAGTGTTCAGC

61 F G L Q A W M E T R P A F D L R K Q L F V W N V I L A V F S

438 CTGTACGGGGGCATCAGGTCCCTGCAGGAACTAATCTACGTGTACACCAACTTCGGCCTGTACTCCACCTACTGCCTCTGCGGCCTTCGT

91 L Y G G I R S L Q E L I Y V Y T N F G L Y S T Y C L C G L R

528 TTCCTTGACAACCGTGTGGGTGGGTTCTGGAACTGGATGTTCACGTTGAGCAAGGTGCCAGAGCTCGGCGACACAGTGTTCATTGTGCTA

121 F L D N R V G G F W N W M F T L S K V P E L G D T V F I V L

618 AGGAAGCAGCCACTAATCTTCCTCCACTGGTACCACCACGTCACTGTCCTCCTCTATGCCTGGTACTCCTACTCCGACTACATCGCCACC

151 R K Q P L I F L H W Y H H V T V L L Y A W Y S Y S D Y I A T

708 GCCCGTTGGTTTGTCTGCATGAACTACCTTGTCCACAGTGCCATGTACAGCTACTACGCCCTCAAGGCCCTCAAGTTCCGGGTTCCCCGC

181 A R W F V C M N Y L V H S A M Y S Y Y A L K A L K F R V P R

798 TGGATTGCCATGAGCATCACCACAGCTCAGTTGGCCCAGATGGTGATGGGAGCAGTAGTAAACATTTGGGCCTACCAGGTGAAACAGGCT

211 W I A M S I T T A Q L A Q M V M G A V V N I W A Y Q V K Q A

888 GGCAATGAGTGTCATGTCTCCTACGATAACATTAAAATCTCCCTCCTCATGTACACATCCTACTTTGTTCTCTTCGCTCGCTTCTTCCGC

241 G N E C H V S Y D N I K I S L L M Y T S Y F V L F A R F F R

978 AAAGCTTATGTTGTGAACCACAAGCAAGGAGGCTCTCAGACACCCAAGGAGTCTATTGCTTATGAAGGGAAAGGTAGCAAGGGTAAACTG

271 K A Y V V N H K Q G G S Q T P K E S I A Y E G K G S K G K L

1068 GAA**TAA**AAGCCTATCTTTACTGGGATTTATAGGGAATAAAACTCATTATTGGTCTGTCAAACACATGGGTCCCACTAATGGCCAATAGCT

301 E

1158 TGCTTGTATCTTACACATGTTTATTTACTACCAGCTGAGGCTATTGCCATATCTAAATTTCACTGTAAAGTATTACAGTACAGTATATGT

1248 ATAAGGAAATGGTATTTTACAGTTTATTCTTTCTTCAGTTTGTCAGTGAATTTTTAACATTTCATCAGTGGTTGCTGACAACAAGAATTA

1338 AGAATTTTGCAATAAAAAAAAAAAAAAAAAAAAAAAAA

**Figure s3** Nucleotide and deduced amino acid sequences of Elovl6c. The nucleotides and amino acids are numbered along the left margin respectively. The start (ATG) and stop (TAA) codons are marked in bold. Membrane-spanning domains are boxed, and endoplasmic reticulum retention signal is bold shaded.
