# Supplementary material for: Cloning and expression characterization of elongation of very long-chain fatty acids protein 6 (elovl6) with dietary fatty acids, ambient salinity and starvation stress in Scylla paramamosain
Source: Front Physiol. 2023 Jul 12;14:1221205. doi: 10.3389/fphys.2023.1221205 (PMC10382226; doi:10.3389/fphys.2023.1221205)
Supplement: Supplementary file 4 [file Table4.DOCX]

**Table s1**

Formulation and proximate composition of the experimental diets (dry matter basis, g kg^-1^)

|  | Diets^1^ |  |  |  |  |  |
| --- | --- | --- | --- | --- | --- | --- |
|  | CFO | SO20 | SO40 | SO60 | SO80 | SO100 |
| Ingredient composition |  |  |  |  |  |  |
| Fish meal | 410 | 410 | 410 | 410 | 410 | 410 |
| Casein | 180 | 180 | 180 | 180 | 180 | 180 |
| Fish oil | 80 | 64 | 48 | 32 | 16 | 0 |
| Soybean oil | 0 | 16 | 32 | 48 | 64 | 80 |
| Dextrin | 220 | 220 | 220 | 220 | 220 | 220 |
| Vitamin mix^2^ | 30 | 30 | 30 | 30 | 30 | 30 |
| Mineral mix^2^ | 20 | 20 | 20 | 20 | 20 | 20 |
| Cholesterol | 8 | 8 | 8 | 8 | 8 | 8 |
| Lecithin | 10 | 10 | 10 | 10 | 10 | 10 |
| Monocalcium phosphate | 15 | 15 | 15 | 15 | 15 | 15 |
| Choline chloride | 7 | 7 | 7 | 7 | 7 | 7 |
| Sodium alga acid | 10 | 10 | 10 | 10 | 10 | 10 |
| Squid Liver Paste | 10 | 10 | 10 | 10 | 10 | 10 |
| Proximate composition (g kg^-1^) |  |  |  |  |  |  |
| Moisture | 99 | 98 | 103 | 102 | 104 | 104 |
| Crude protein | 457 | 455 | 458 | 455 | 453 | 449 |
| Crude lipid | 95 | 99 | 94 | 101 | 96 | 97 |
| Ash | 110 | 109 | 104 | 104 | 106 | 108 |

^1^ CFO means that fish oil is the only dietary lipid source, numerical values after SO refer to the percentage of dietary fish oil replaced by soybean oil.

^2^ Xingmuwei Animal Health Product, Xiamen, Fujian, China.

**Table s2**

Fatty acid composition (% total fatty acids) of experimental diets.

| Fatty acid^1^ | Diets^2^ |  |  |  |  |  |
| --- | --- | --- | --- | --- | --- | --- |
|  | CFO | SO20 | SO40 | SO60 | SO80 | SO100 |
| 14:0 | 3.2 | 2.6 | 2.2 | 1.8 | 1.4 | 0.6 |
| 16:0 | 15.2 | 14.5 | 13.8 | 13.3 | 12.1 | 11.6 |
| 18:0 | 4.4 | 4.5 | 4.7 | 4.8 | 4.9 | 4.9 |
| 20:0 | 1.4 | 1.3 | 1.1 | 0.9 | 0.8 | 0.7 |
| 22:0 | 0.7 | 0.6 | 0.5 | 0.5 | 0.3 | 0.2 |
| 16:1n-7 | 3.9 | 3.2 | 2.8 | 2.2 | 1.3 | 0.7 |
| 18:1n-9 | 19.2 | 22.3 | 24.1 | 26.2 | 28.4 | 29.7 |
| 20:1n-9 | 1.5 | 1.3 | 1.2 | 1.2 | 1.2 | 1.1 |
| 22:1n-11 | 0.4 | 0.4 | 0.3 | 0.2 | 0.2 | 0.1 |
| 18:2n-6 | 19.1 | 25.0 | 30.2 | 34.5 | 37.6 | 41.6 |
| 18:3n-6 | 1.7 | 1.4 | 1.0 | 0.8 | 0.6 | 0.4 |
| 18:3n-3 | 3.0 | 3.3 | 3.8 | 4.1 | 4.5 | 4.9 |
| 20:2n-6 | 0.9 | 0.8 | 0.5 | 0.4 | 0.4 | 0.2 |
| 20:3n-3 | 3.2 | 2.5 | 2.0 | 1.5 | 0.9 | 0.5 |
| 20:4n-6 | 1.0 | 0.8 | 0.7 | 0.6 | 0.3 | 0.1 |
| 20:5n-3 | 9.1 | 6.7 | 4.5 | 3.3 | 2.3 | 1.1 |
| 22:6n-3 | 11.9 | 8.8 | 6.4 | 3.7 | 2.8 | 1.5 |
| ΣSFA^3^ | 24.9 | 23.5 | 22.3 | 21.3 | 19.5 | 18 |
| ΣMUFA^4^ | 25.0 | 27.2 | 28.4 | 29.8 | 31.1 | 31.6 |
| ΣPUFA^5^ | 24.7 | 30.5 | 35.5 | 39.8 | 43.1 | 47.1 |
| ΣHUFA^6^ | 25.2 | 18.8 | 13.6 | 9.1 | 6.3 | 3.2 |
| Σn-3 FA^7^ | 27.2 | 21.3 | 16.7 | 12.6 | 10.5 | 8 |
| Σn-6 FA^8^ | 22.7 | 28.0 | 32.4 | 36.3 | 38.9 | 42.3 |
| n-3/n-6 | 1.2 | 0.8 | 0.5 | 0.3 | 0.3 | 0.2 |

^1^ Data expressed as area percentage of fatty acid methyl ester, represent mean ± SEM, n = 3. Fatty acids present at ≤ 0.1 percentage of total fatty acids are not included.

^2^ CFO means that fish oil is the only dietary lipid source, numerical values after SO refer to the percentage of dietary fish oil replaced by soybean oil.

^3^ SFA: 14:0, 16:0, 18:0, 20:0, 22:0.

^4^ MUFA: 16:1n-7, 18:1n-9, 20:1n-9, 22:1n-11.

^5^ PUFA: 18:2n-6, 18:3n-6, 18:3n-3, 20:2n-6.

^6^ HUFA: 20:3n-3, 20:4n-6, 20:5n-3, 22:5n-3, 22:6n-3.

^7^ n-3 FA: 18:3n-3, 20:3n-3, 20:5n-3, 22:6n-3.

^8^ n-6 FA: 18:2n-6, 18:3n-6, 20:2n-6, 20:4n-6.
